# ATP6V1H Deficiency Impairs Bone Development through Activation of MMP9 and MMP13

**DOI:** 10.1371/journal.pgen.1006481

**Published:** 2017-02-03

**Authors:** Yihan Zhang, Haigen Huang, Gexin Zhao, Tadafumi Yokoyama, Hugo Vega, Yan Huang, Raman Sood, Kevin Bishop, Valerie Maduro, John Accardi, Camilo Toro, Cornelius F. Boerkoel, Karen Lyons, William A. Gahl, Xiaohong Duan, May Christine V. Malicdan, Shuo Lin

**Affiliations:** 1 Laboratory of Chemical Genomics, School of Chemical Biology and Biotechnology, Peking University Shenzhen Graduate School, Shenzhen China; 2 Department of Molecular, Cell, and Developmental Biology, University of California Los Angeles, Los Angeles, California, United States of America; 3 Department of Orthopaedic Surgery and Orthopaedic Institute for Children, David Geffen School of Medicine, University of California, Los Angeles, California, United States of America; 4 Medical Genetics Branch, National Human Genome Research Institute, NIH, Bethesda, Maryland, United States of America; 5 NIH Undiagnosed Diseases Program, Common Fund, Office of the Director, NIH and National Human Genome Research Institute, NIH, Bethesda, Maryland, United States of America; 6 Zebrafish core, Translational and Functional Genomics Branch, National Human Genome Research Institute/NIH, Bethesda, Maryland, United States of America; 7 Office of the Clinical Director, National Human Genome Research Institute/NIH, Bethesda, Maryland, United States of America; 8 State Key Laboratory of Military Stomatology, National Clinical Research Center for Oral Diseases, Shaanxi Key Laboratory of Oral Diseases, Department of Oral Biology, Clinic of Oral Rare and Genetic Diseases, School of Stomatology, The Fourth Military Medical University, Xi’an, China; Stanford University School of Medicine, UNITED STATES

## Abstract

ATP6V1H is a component of a large protein complex with vacuolar ATPase (V-ATPase) activity. We identified two generations of individuals in which short stature and osteoporosis co-segregated with a mutation in *ATP6V1H*. Since V-ATPases are highly conserved between human and zebrafish, we generated loss-of-function mutants in *atp6v1h* in zebrafish through CRISPR/Cas9-mediated gene knockout. Homozygous mutant *atp6v1h* zebrafish exhibited a severe reduction in the number of mature calcified bone cells and a dramatic increase in the expression of *mmp9* and *mmp13*. Heterozygous adults showed curved vertebra that lack calcified centrum structure and reduced bone mass and density. Treatment of mutant embryos with small molecule inhibitors of MMP9 and MMP13 significantly restored bone mass in the *atp6v1h* mutants. These studies have uncovered a new, ATP6V1H-mediated pathway that regulates bone formation, and defines a new mechanism of disease that leads to bone loss. We propose that MMP9/MMP13 could be therapeutic targets for patients with this rare genetic disease.

## Author Summary

Osteoporosis, a major health problem worldwide, is characterized by low bone mineral density (BMD) and a propensity to fracture. Genetic factors are clearly the major determinants of BMD, but their identification and contribution to osteoporosis risk have been difficult to assess in humans. Genome-wide association studies (GWAS) have identified numerous sequence variants that influence BMD. The loci identified to date, however, account for only a small fraction of the total variation in BMD. Through analysis of a pedigree with an undiagnosed disease in which affected members have markedly low bone mass, we identify a novel and critical bone formation pathway, mediated through the gene ATP6V1H. Zebrafish lacking apt6vh1 demonstrate loss of bone mass, and exhibit increased mmp9 and mmp13 levels; inhibition of mmp9 and mmp13 led to the rescue of bone density defects. Here we show that happloinsufficiency of ATP6V1H is associated with osteoporosis in both humans and zebrafish. This study exemplifies the value of studying rare diseases to understand prevalent ones.

## Introduction

ATP6V1H is a member of the family of vacuolar ATPases (V-ATPases), and a component of protein complexes responsible for acidification of intracellular compartments in all eukaryotic cells [1]. Specifically, V-ATPases regulate protein degradation, mediate pH homeostasis, and facilitate cellular function and development through acidification. In human, mutations in *ATP6B1*, which encodes the β-subunit of the apical proton pump mediating distal nephron acid secretion, cause distal renal tubular acidosis, a condition characterized by impaired renal acid secretion, resulting in metabolic acidosis [2]. In osteoclasts, V-ATPases acidify the space that is in contact with the bone surface; this process is essential for bone resorption. Consequently, components of V-ATPases are potential therapeutic targets for the treatment of osteolysis [3]. Moreover, *Atp6i*-deficient mice exhibit severe osteopetrosis due to loss of osteoclast-mediated extracellular acidification; the osteopetrotic phenotype may be due to failure of osteoclast formation during development [4]. Using zebrafish model and patient phenotypic data, we show that haploinsufficiency of the V1H subunit of V-ATPases can result to osteoporosis.

Through the NIH Undiagnosed Diseases Program (UDP) [5–7], we identified a 48-year old woman and her 24-year old son who exhibited markedly low bone density. This phenotype segregated with a monoallelic mutation in *ATP6V1H*. Since osteoporosis is the opposite of the osteopetrosis seen in *Atp6i*-deficient mice and expected for reduced V-ATPase activity, we studied the phenotype of zebrafish harboring a null mutation in *atp6v1h*. Indeed, this animal model displayed reduced bone mass, and we demonstrated that the bone defect was mediated by increased activity of mmp9 and mmp13. Small molecule inhibitors of both proteins notably restored bone volume, suggesting that MMP9 and MMP13 could be therapeutic targets for treatment.

## Results

### Patients

**Fig 1:**
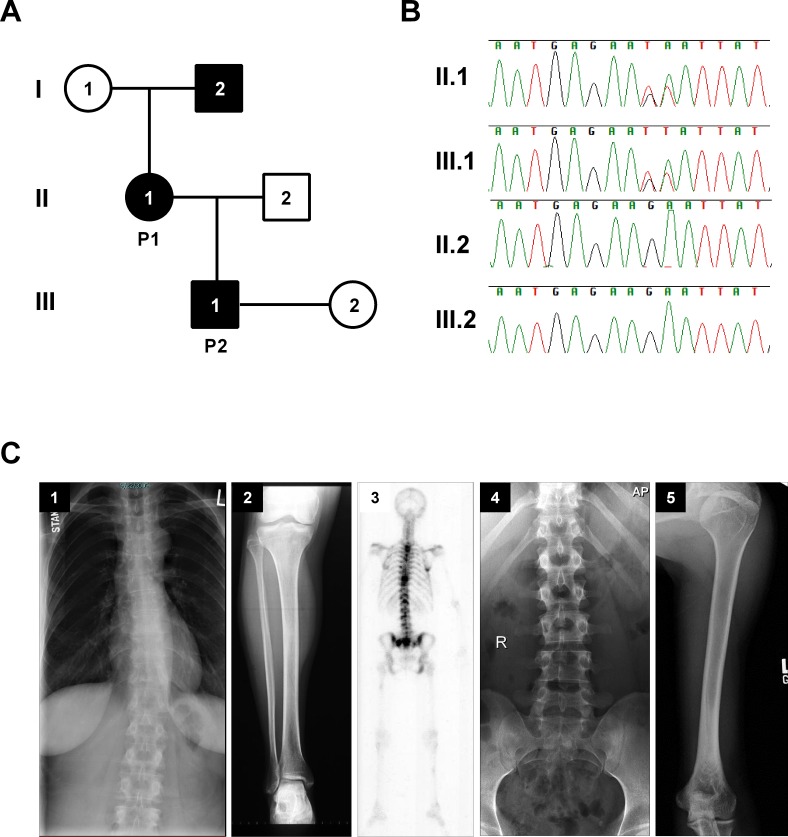
Clinical features of patients with *ATP6V1H* mutations. The family comes from a three-generation pedigree; affected members are shaded in black (**A**); Chromatograms showing that the *ATP6V1H* mutation is present in affected family members, (II.1 and III.1 (**B**); DNA from I.1 is not available; (C) Radiographs (**C**) from Patient 1 (P1) (1, 2, and 3) and Patient 2 (P2) (4, and 5) reveal that P1 has scoliosis (C1) and increased radiolucency in tibia (C2). Bone scan shows multiple sites of increased bone mineral density uptake–tip of right scapula and multiple areas of thoracic and lumbar spine, sacrum, both sacroiliac joints, left humeral head, shaft of left femur, right tibia and right ribs (C3). Some increase in radiolucency especially in the lumbar spine (C4), is seen in P2.

Patient 1 (P1; II.1 in Fig 1A) is a 48-year old woman and Patient 2 (P2; III.1 in Fig 1A) is her 24-year old son. Both exhibited short stature, bone pain and decreased bone density (Fig 1C, 1–5 and S1A Fig). The father of P1 (I.2 in Fig 1C) reportedly had the same features, with dilated frontal sinuses and decreased bone density (S1B and S1C Fig). Exome sequencing revealed NM_015941.2:c.1158_1159delinsTT (p.Lys386_Asn387delinsAsnTyr, named K386N/N387Y hereafter) in *ATP6V1H* that segregated with the osteoporotic phenotype (Fig 1B). *In silico* analysis for protein stability predicted that both amino acid changes due to the mutation (K386N and N387Y) can alter protein stability (I-Mutant2.0). To confirm this finding, we generated a stable cell line expressing K386N/N387Y and measured protein half-life. After protein synthesis inhibition by cycloheximide treatment, the mutant protein decayed rapidly compared to the wild-type protein, suggesting that the mutation produced an unstable protein (S2 Fig).

### Bone but not cartilage is defective in *atp6v1h*-deficient zebrafish

**Fig 2:**
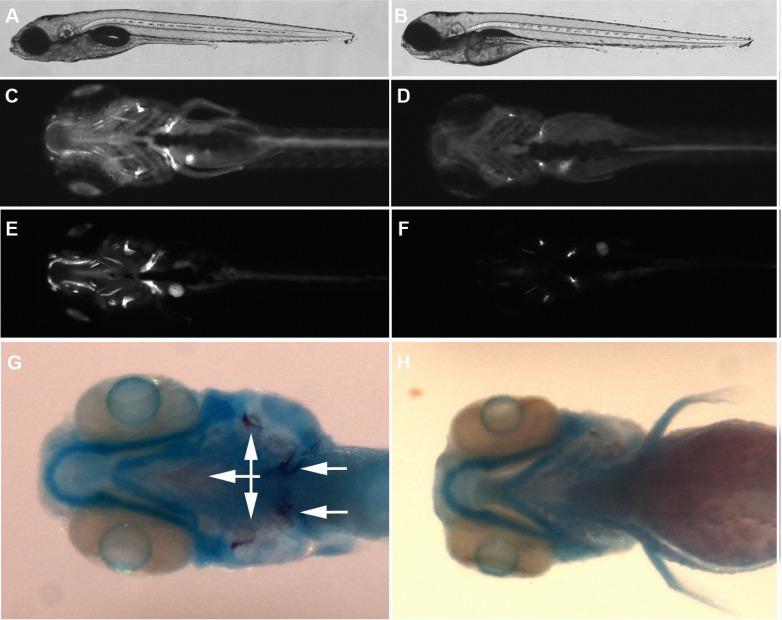
Bone but not cartilage cells are defective in *atp6v1h*-deficient zebrafish. (**A-B**) shows an image of a 6 days post fertilization (dpf) wild type and mutant embryo with relatively normal gross morphology. (**C-F**) Day 6 and 8 wild type and mutant embryos showing reduction in bone detected by calcein staining (ventral view, bright fluorescent stains indicate calcified bone). In (**G-H**), double staining for cartilage and bone demonstrate reduction of mineralized bone (dark purple, arrows) but not cartilage (blue). A, C, E and G are wild type. B, D, F, and H are mutant.

Based on the finding that K386N/N387Y mutant protein was unstable, we reasoned that loss of function mutation in an animal model could address the function of *ATP6V1H*. We obtained a null mutation of *atp6v1h* in zebrafish through CRISPR/Cas9 mediated gene knockout (S3 Fig and supplementary information, designated as *atp6v1h*^*la439*^). Homozygous mutants died at 10–11 days post fertilization (dpf). Up to dpf 6, the mutant embryos did not appear to have major abnormalities in morphology, but they failed to inflate their swim bladders (Fig 2). Although a retrovirus-induced insertional mutation of *atp6v1h* has been previously reported for zebrafish, no bone related phenotypes were analyzed [8]. In this study we showed that *atp6v1h* expression was largely confined to the head region, where cartilage and bone cells first develop, and this expression was completely lost in our mutant (S3 Fig), suggesting true loss of function. Analysis by calcein staining indicated that *atp6v1h* -/- embryos had greatly reduced or absent calcification of bone cells (Fig 2). Co-staining of bone and cartilage cells showed that cartilage cells were largely unaffected, while mineralized bone was nearly absent (Fig 2). We did not observe any obvious skeletal defects in wild type (+/+) or heterozygous (+/-) embryos, suggesting that complete loss of function for *atp6v1h* is required to produce the observed embryonic phenotype.

We next determined if the *ATP6V1H* K386N/N387Y mutation had a dominant effect on zebrafish development. The affected region of the protein is fully conserved in zebrafish *atp6v1h* (S3 Fig). We generated cDNA constructs containing the same mutation or the wild type sequence as a control to evaluate the effects of the mutation. We next restored bone mineralization in the mutant zebrafish by injecting with wild type mRNA; injecting K386N/N387Y mutant mRNA did not restore mineralization (S5 Fig), suggesting that the change leads to a loss-of-function mutation. Additionally, injection of wild-type embryos with K3866N/N387Y mRNA had no obvious effect on embryonic development, suggesting that this mutation did not result in gain-of-function S5 Fig).

**Fig 3:**
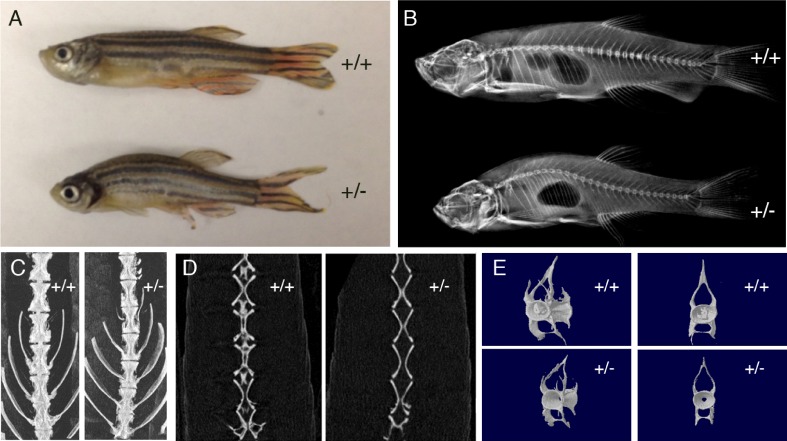
Micro-computed tomography scan of adult zebrafish. Adult zebrafish heterozygous for atp6V1h mutation (+/-) show curved body, compared to the wild type (+/+) sibling (**A**). X-ray of the same +/- fish shows curved spine compared to the +/+ sibling (**B**). Vertebra of +/- fish has bristlier surface and smaller size (**C**). Vertebra of +/- fish lacks calcified structure in the centrum cavity (**D**). Individual vertebra of +/- fish shows bristlier surface and smaller size with a hollow centrum (**E**) (n = 3 each).

We then analyzed the phenotype of adult fishes that lacked *atp6V1h* in one allele (heterozygous, +/-). By eight months after birth, over 50% of the heterozygous adults showed a curved body (Fig 3). X-ray and micro-CT studies revealed that bone mineral density, bone volume and bone surface were all reduced but the later two reductions were more dramatic (S6 Fig). The most remarkable discover is that the vertebrae of heterozygous fish completely lacked calcified structure in the centrum cavity (Fig 3). Additionally, overall skeleton structures appeared smaller and had subtle but notable abnormalities. Furthermore, this adult phenotype was observed in the previously reported retroviral insertion mutant allele of zebrafish *atp6v1h* gene (S6 Fig) [8].

### *mmp9* and *mmp13* are highly induced by atp6v1h-deficiency

**Fig 4:**
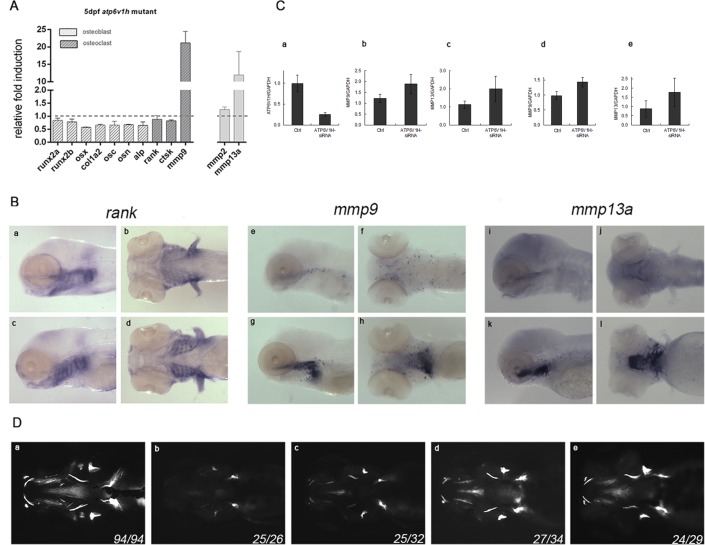
Induction of *mmp9/mmp13* and rescue of atp6v1h-deficient phenotype. In (**A**), qPCR analysis of designated marker genes showing significant induction of *mmp9* and *mmp13* mRNA expression in *atp6v1h* mutant. Analysis of osteoclast cells by RNA whole mount *in situ* hybridization. *atp6v1h* mutant embryos showing significant increases of mmp9 and mmp13a signals in embryonic head skeleton (e/f/i/j wild type, g/h/k/l mutant), but osteoclast marker *rank* pattern is not changed (a/b wild type, c/d mutant) (**B**). In (**C**), analysis of *Mmp9/Mmp13* expression in mouse osteoclast Raw264.7 cells after knockdown of *Atp6v1h* by siRNA is shown. When *Atp6v1h* expression was knocked down by siRNA (a), the expression of *Mmp9* (b) and *Mmp13* (c) were induced. Similarly, the induction of *Mmp9* (d) and *Mmp13* (e) was also observed in RANKL-induced differentiated mouse osteoclast Raw264.7 cells. In (**D**), MMP inhibition rescues the bone phenotype in *Atp16V1h* mutant fish as detected by calcein staining; a, wild type, 0.5% DMSO; b, mutant, 0.5% DMSO; c, mutant treated with 200uM MMP9 inhibitor II in 0.5% DMSO; d, mutant treated with 20uM MMP13 inhibitor in 0.5% DMSO; e, mutant treated with 50uM MMP9/13 inhibitor I in 0.5% DMSO. Images are ventral views of 6 dpf embryos.

To determine the molecular mechanism underlying the bone abnormalities associated with *atp6v1h* deficiency, we analyzed lineage-specific markers involved in different stages of osteoblast and osteoclast development. First, we determined if *atp6v1h* was expressed in osteoblast or osteoclast cells of zebrafish by performing high resolution double fluorescent *in situ* hybridization using *atp6v1h* probe with the osteoblast marker *runx2a* or osteoclast marker *rank*. This analysis showed that *atp6v1h* had broader co-expression with osteoclast marker *rank* but less with osteoblast marker *runx2a* (S4 Fig), which is consistent with the previous report that V-ATPase is enriched in osteoclast cells [1]. Quantitative PCR (qPCR) analysis was further performed to determine mRNA expression of major bone-specific marker genes, including *runx2*, *osx*, *col1a2*, *osc*, *osn* and *alp* for osteoblasts and *rank*, *cstk* and *mmp9* for osteoclasts between homozygous mutant and sibling embryos. The expression of intermediate and late osteoblast genes in the *atp6v1h*-deficient zebrafish was marginally reduced (Fig 4A). RNA *in situ* hybridization (S7 Fig), showing a slight reduction in the expression of the osteoblast genes, confirmed the qPCR data. The expression of the osteoclast genes *rank* and *cstk* did not obviously differ from wild type, but *mmp9* mRNA levels were vastly elevated in the *atp6v1h*-deficient zebrafish (Fig 4A). *In situ* hybridization demonstrated that the pattern and level of expression of the osteoclast marker *rank* were similar in the mutant and wild type zebrafish, and that the increased *mmp9* mRNA expression occurred primarily in the head region of the zebrafish (Fig 4B), where *mmp9* was previously identified to be expressed as an osteoclast marker [9,10]. Considering that we mainly detected *mmp9* up-regulation in ATP6V1H-defficient mammalian osteoclast but not in osteoblast and chondrocytes (Fig 4C and S8 Fig), we hypothesize that *mmp9* is mainly up-regulated in zebrafish osteoclasts. It remains to be determined if other cell types are also induced to express excess amount of *mmp9*.

The finding that *mmp9* was up-regulated prompted us to analyze *mmp2* and *mmp13*, previously reported to be involved in *mmp9* related bone homeostasis [9,10]. Expression of *mmp13a* mRNA was significantly increased in atp6v1h-deficient zebrafish embryos, but that of *mmp2* was not (Fig 4A). Zebrafish also have a gene named *mmp13b* that appears to be a pseudogene since there was no detectable expression by qPCR either in the wild type or atp6v1h-deficient embryos. Further analysis of *mmp13a* expression by RNA *in situ* hybridization revealed a pattern and intensity that was very similar to that of *mmp9* (Fig 4B).

To determine if *Mmp9* and *Mmp13* expression was similarly induced in mammalian cells deficient of *Atp6v1h*, we performed siRNA knockdown experiments in mouse osteoclast, osteoblast and chondrocyte cell types. We found that both *Mmp9* and *Mmp13* were induced when *Atp6v1h* was knocked down in mouse osteoclast cells, but reduced in osteoblast and chondrocyte (Fig 4C and S8 Fig), suggesting a conserved action of Atp6v1h deficiency. To study if maturation was affected we performed TRAP staining on these osteoclast cells. This study revealed more TRAP staining in ATP6V1H deficient cells (S9 Fig). ATP6V1H siRNA groups have 0.41%±0.07 TRAP positive cells whereas scrambled controls have 0.13%±0.01 (S2 Table). Consistent to this finding, we found adult zebrafish +/- for *atp6v1h* appeared to have more TRAP staining in bone sections (S10 Fig). Overall, these data suggest that a higher osteoclast activity is induced by atp6v1h deficiency.

### MMP9 and MMP13 inhibitors rescue bone abnormalities in atp6v1h mutants

Because abnormal expression of *mmp9* and *mmp13* occurs in human diseases such as cancer, arthritis and cardiovascular disorders, small molecule inhibitors specific to MMP9 and MMP13, respectively, or to both MMP9 and MMP13 were available [11,12]. We treated *atp6v1h* mutant zebrafish embryos with these inhibitors and found that an MMP13 inhibitor restored bone formation nearly toward normal while an MMP9 inhibitor had a reduced rescue effect (Fig 4D). Treatment with an inhibitor that has dual activity on both MMP9 and MMP13 rescued the bone phenotype to an extent similar to the inhibitor of MMP13 (Fig 4D). These findings suggest that increased *mmp9* and *mmp13* expression contribute significantly to the bone defect, and targeting MMP13 would likely alleviate this problem.

## Discussion

We describe the consequences of *atp6v1h* mutation in zebrafish, based upon the ascertainment of a family with 3 generations of osteoporosis segregating with a K386N/N387Y mutation in *ATP6V1H*. We discovered a profound reduction in the amount of mature bone in homozygous *atp6v1h* zebrafish mutants; cartilage formation was largely unaffected. Since injection of mRNA carrying the human point mutation failed to induce any abnormality in wild type embryos, we propose that K386N/N387Y is a loss-of-function mutation. Given that adult zebrafish heterozygous for *atp6v1h* deficiency has bone defects and the human mutation was transmitted in a heterozygous manner, this mutation can lead to haploinsufficiency of ATP6V1H protein in humans.

The cellular targets of ATP6V1H function remain to be determined. Osteoblast markers were only marginally affected, suggesting that ATP6V1H does not exert its primary effects on osteoblast formation. The skeletal abnormalities in *atp6v1h* mutant zebrafish were associated with dramatically increased expression of *mmp9* and *mmp13*, and inhibition of MMP9 and MMP13 significantly restored bone volume. MMP9 and MMP13 are members of the matrix metalloproteinase family, which are important for remodeling of the extracellular matrix in diverse biological processes. The functions of MMP2, MMP9 and MMP13 in skeletal remodeling have been extensively investigated through genetic studies. While *Mmp2*-null mice have osteoporosis [13], *Mmp9*-null and *Mmp13*-null mice exhibit enlarged hypertrophic zones and osteopetrosis [14,15]. The double knockout of *Mmp9* and *Mmp13* leads to a more dramatic expansion in the length of the hypertrophic zone than in single knockouts, suggesting a synergistic relationship between *Mmp9* and *Mmp13 [16]*. This is consistent with the findings in our *atp6v1h* zebrafish mutant, in which increased *mmp9* and *mmp13* expression combined to induce the bone defect.

MMP13 acts as a collagenase to degrade type 1 collagen, the main component of bone extracellular matrix. Collagenases degrade the osteoid layer covering the surfaces of bone to expose calcified bone matrix to osteoclasts [17–20]. Significant induction of *mmp13* in *atp6v1h* zebrafish mutant might, therefore, accelerate the degradation of type 1 collagen and provide more mineral contact for osteoclasts, without increasing osteoclast numbers (Fig 4B). Moreover, extracellular matrix has been demonstrated to play a role in osteoblast growth and differentiation [21]. Type I collagen influences osteoblast development and maintenance by modifying the expression of genes supporting cell growth, adhesion and extracellular matrix mineralization [21]. Thus, enhanced degradation of matrix collagen leads not only to increased bone resorption but also to suppression of bone formation [22]. This is consistent with our findings that expression of genes that label intermediate and mature stage osteoblasts is reduced relative to early markers of osteoblast formation (Fig 4).

In zebrafish and mammals, *mmp9* is highly expressed in skeletal cells, especially in osteoclasts [9,23–25]. Mmp9 can degrade denatured collagens. Therefore, increased *mmp9* expression in *atp6v1h* mutant zebrafish would be predicted to contribute to ECM degradation. Since the expression level of other osteoclast genes, *rank* and *ctsk*, remained largely unchanged, we speculate that increased osteoclast numbers do not play a significant role in reducing bone density in *atp6v1h* mutants. However, increased maturation and activity of osteoclasts might be more relevant, as demonstrated by the increased differentiation revealed by TRAP staining in mouse ATP6V1H deficient pre-osteoclast cells and adult heterozygous *atp6v1h* zebrafish.

Apart from matrix degradation, Mmp9 also promotes the apoptosis of hypertrophic chondrocytes in the growth plate [14]. Hence, increased MMP9 activity in *atp6v1h* mutants may also be associated with accelerated loss of hypertrophic chondrocytes or abnormal apoptosis, contributing to bone loss. Interestingly, Mmp13 and Mmp9 induction are also found in patients with arthritis [26]; these individuals also have abnormal matrix, and could share the same pathogenetic mechanism. Inhibition of MMP9 and MMP13 might be considered as a therapeutic approach for treating these diseases.

Vacuolar ATPases have complicated structures and functions, which may explain why mutations in some subunits cause osteopetrosis while mutations in others cause osteoporosis. V-ATPase is composed of 14 different subunits arranged in two structural domains, V1 and V0. ATP hydrolysis occurs in the cytoplasmic V1 domain whereas proton translocation occurs through the membrane V0 domain [1]. Knockout of the a3 subunit of V0 directly blocks its function in proton translocation, thus causing an acidification defect; *Atp6i*-deficient mice exhibit severe osteopetrosis [2]. Subunit H of V1 activates ATP-driven proton pumping in the intact V-ATPase complex but, when V1 and V0 are disassociated, the H subunit inhibits the Mg-ATPase activity of the cytosolic V1 domain to prevent unnecessary ATP hydrolysis [27,28]. Isolated V1 complexes without subunit H show significantly higher levels of ATPase activity compared to the wild-type V1 complexes. Therefore, in addition to the acidification defect caused by loss of the H subunit, ATP hydrolysis is increased [29]. In yeast strains lacking subunit H, V1-V0 complexes are inactive and unstable, suggesting that the H subunit serves as an activator of the intact V-ATPase [30].

**Fig 5:**
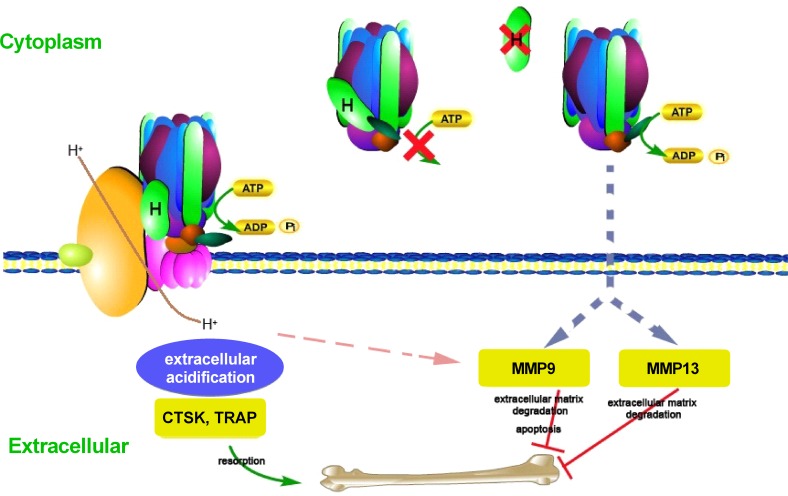
Model of action. A schema showing Vacuolar ATPase and its subunits. Subunit H (V1, cytosolic domain) is shown in green. Under physiologic conditions, ATP hydrolysis occurs in the cytosolic domain while proton translocation occurs through the membrane domain. The V1H subunit activates ATP-driven proton pumping in the intact V-ATPase complex, but when V1 and V0 are disassociated, inhibition of the Mg-ATPase activity of the V1 domain occurs to prevent unnecessary ATP hydrolysis. When V1H is mutated, significantly higher levels of ATPase activity compared to the wild-type V1 complexes is expected, causing increased matrix degradation, leading to decreased bone density via increased MMP9/MMP13 activity.

We propose a model of action in which mutation of the V0 subunit a3 causes reduced acidification, increasing bone density whereas mutation of the V1 subunit H causes excessive ATP hydrolysis, decreasing bone density through increased MMP9/MMP13 activity (Fig 5). Supporting this hypothesis is that knockdown of *Atp6i* in B16-F10 cells causes decreased expression of *Mmp9* [31]. In our model, although acidification by V-ATPase is marginalized when *ATP6V1H* is mutated, the increase in MMP9 /MMP13 activity related to the constitutive Mg-ATPase activity of the cytosolic V1 sector, dominates, resulting in a net reduction in bone density. Enhanced ATP hydrolysis by cytosolic Mg-ATPase activity might activate signaling pathways to stimulate *mmp9* and *mmp13* expression, a topic for future research. In any event, the bone defect caused by *atp6v1h* deficiency through activation of *mmp9/13* represents a novel finding that offers potential therapeutic entry points for related diseases, since MMP9 levels have been correlated to severity of osteoporosis [32].

## Materials and Methods

### Ethics statement

Patients were enrolled in the NIH Undiagnosed Diseases Program [5–7] and in protocol 76-HG-0238, “Diagnosis and Treatment of Patients with Inborn Errors of Metabolism and Other Genetic Disorders”, approved by the NHGRI Institutional Review Board. All patients involved in this study gave written, informed consent. All zebrafish experiments were approved by the UCLA Animal Care and Use Committee and the NHGRI ACUC Committee.

### Zebrafish

To establish a mutant *ATP6V1H* zebrafish line, gRNA targeting the fourth exon of ATP6V1H was designed and shown to induce indels by injection with Cas9 mRNA in zebrafish. Several mutant lines were established using gRNA with the sequence 5’-GTGTGTCATCAATCAGGGTCAGG; the line used in this study carried a 17 bp insertion and produced a truncated peptide of 94aa, whereas the wild type ATP6V1H encodes a 463aa protein. Stable heterozygous carriers of the mutation were mated to produce homozygous mutants. Details of mutation generation and characterization are presented in the supplementary section and S3 Fig. Staining for bone and cartilage cells was performed as described [33,34].

### Quantitative PCR

Total RNA was extracted in Trizol Reagent (Life Technologies) according to the manufacturer’s protocol. cDNA was synthesized using Superscript III (Life Technologies) and oligodT (Life Technologies) following the manufacturer’s protocol. Quantitative real-time PCR was performed using FastStart SYBR Green Master (Roche). Primer sequences are listed in S1 Table. Values of three independent samples (n = 50 each sample) are shown.

### siRNA knockdown of *Atp6v1h* in mouse cells

See supplementary materials.

### Whole-mount in situ hybridization

The probes listed in the figures were generated by PCR using zebrafish embryonic cDNA (Primers in S1 Table). Regular and double fluorescence whole-mount in situ hybridization was, respectively, performed as previously described [35,36].

### Micro-computed tomography analysis and TRAP staining in adult zebrafish

For X-ray imaging, adult zebrafish were fixed in 10% neutral-buffered formalin for 24 hours and X-ray images were taken using Faxitron MX-20 System Digital Radiography system (Faxitron Bioptics, Tucson, Arizona). For micro-computed tomography (μ-CT), adult zebrafish were fixed in 10% neutral-buffered formalin for 24hours and mounted in 1% low-melt agarose (Sigma) in a plastic vial. Specimens were scanned using SkyScan1172 Micro-CT scanner (Bruker MicroCT, Kontich, Belgium) with the X-ray power at 55kVp and 185μA, and specimens were scanned at a 10 μm voxel resolution. A three-dimensional reconstruction was generated with NRecon software from the set of scans. The images were then analyzed using CTAn (v.1.14.4) and CTVol (v.2.2) softwares. The first caudal bones were assessed for bone mineral density (BMD), bone volume (BV), and bone surface (BS). For TRAP staining, fixed fish were decalcified and sectioned (5 um) after paraffin embedding. TRAP staining was performed using a kit (Sigma 387A-KT) according to the manufacturer's instructions.

### Treatment with MMP inhibitors

MMP9 Inhibitor II (Cat# 444293), MMP13 Inhibitor (Cat# 444283) and MMP9/13 Inhibitor I (Cat# 444252) were purchased from EMD Millipore. Zebrafish were treated with designated concentrations of each inhibitor from 1 to 6 days post fertilization in 2 ml of fish water.

## Supporting Information

S1 Fig Additional clinical features. ATable shows the levels of biochemistry findings from Patient 1 (P1) and Patient 2 (P2), and Bone mineral densitometry scores.BRadiographic pictures from I.2 (father of P1) showing scoliosis and fractured fibula.CDual-energy x-ray absorptiometry (DXA) results of I.2.
(TIF)

S2 Fig Mutant ATP6V1H protein is degraded more rapidly than wild type protein. Cell lysates form HEK cells transfected with full length *ATP6V1H* wild-type or mutant (c.1158_1159delinsTT) cDNA and treated with cycloheximide were subjected to western blot. Figure shows ATP6V1H bands at 0 hour (baseline), 6 hours, 12 hours, and 24 hours. Protein loading is normalized with GAPDH. Experiments were done in three replicates.
(TIF)

S3 Fig Generation and Genotyping of *atp6v1h* zebrafish mutants. Protein alignment of ATP6V1H in human (NP_057025.2) and zebrafish (NP_775377.1) shows high homology (~85%). More importantly, the region where the mutation is located is highly conserved (**A**). Using CRISPR/Cas9, guide RNA (gRNA) targeting exon 4 of zebrafish *atp6v1h* was co-injected with Cas9 mRNA into zebrafish embryos and detected by T7 endonuclease digestion. Sequence of gRNA and target is shown (**B**). Several founders with indels were screened for germline transmission, and the one with 17bp insertion was used throughout the study; The 17bp insertion is predicted to produce a termination codon 94 amino acids after initiation of start codon (**C**). Genotyping done by polyacrylamide gel electrophoresis (PAGE) analysis demonstrating a 168 bp band for wild type allele, and 185 bp band for mutant allele; representative results for wild type (+/+), heterozygous (+/-), and homozygous (-/-) embryos are shown (**D**). Using probes specific for *atp6v1h*, RNA *in situ* hybridization analysis of *atp6v1h* showed expression in the head region in wild type embryos, while the expression is nearly absent in homozygous *atp6v1h* embryos, suggesting that the 17bp insertion may lead to nonsense-mediated decay (NMD) of mRNA and result in loss-of-function of this gene (**E**).
(TIF)

S4 Fig Co-expression analysis of atp6v1h with osteoclast or osteoblast markers. Double fluorescence RNA whole mount *in situ* hybridization with *atp6v1h* and *rank or runx2a* probes were performed on 60 hpf embryos and imaged by confocal microscopy. Upper panels are ventral views of merged lower magnification images of *runx2a* and *atp6v1h* or *rank* and *atp6v1h*, in which the boxes indicate the regions shown in the lower panels of higher magnification. The lower panels are images of individual colors (green fluorescence for probes of *runx2a* or *rank*, red fluorescence for *atp6v1h*) and merged color (*runx2a* + *atp6v1h*, *rank*+ *atp6v1h*). Arrowheads point to cells that are mainly positive for runx2a and arrows point to cells that appear positive both for *rank* and *atp6v1h* (co-expression: yellow fluorescence).
(TIF)

S5 Fig Rescue of *atp6v1h* mutant zebrafish. Bone staining of uninjected wild type and *atp6v1h* -/- embryos; loss of bone mineralization is seen in *atp6v1h* mutant embryos (A and B). *atp6v1h* mutant embryos at single-cell stage injected with wild type mRNA (C, 200 pg; D, 300 pg) were stained at 5dpf; representative figures demonstrate the rescue of bone phenotype, seen as increase in bone mineralization. *atp6v1h* mutant embryos injected with mutant mRNA containing the same K386N and N387Y mutations found in humans (E, 200 pg; F, 300 pg) at single-cell stage and stained at 5 dpf; representative figures show the absence of bone staining, indicating that the mutations create a non-functional gene. Wild type and mutant mRNA were injected into wild type embryos at single-cell stage and stained at 5 dpf (G and H); representative figures show no alteration in morphology and bone staining, suggesting the absence of gain-of-function resulting from overexpression of either wild type or mutant mRNA.
(TIF)

S6 Fig Micro-CT analysis of heterozygous adult bone phenotype. A. Micro-CT data of the first to fifth caudal vertebrae. Wild type sibling +/+. Heterozygous +/-. BMD, BV and BS designate bone mineral density, bone volume and bone surface (n = 3). B. Micro-CT images of bone in 8-month-old male adult wild type zebrafish and heterozygous allele of retrovirus insertion mutant, *atp6v1h*^*hi923+/-*^ (n = 5 each).
(TIF)

S7 Fig RNA *in situ* hybridization analysis of osteoblast and osteoclast gene markers. *In situ* hybridization markers of osteoblast development (*runx2a*, *runx2b*, *osx*, *osn*, *col1a2*) show slight reduction between 5dpf siblings (wild type) or mutant (-/-).
(TIF)

S8 Fig siRNA knockdown of *Atp6v1h* in mouse osteoclast, osteoblast and chondrocyte cells. siRNA knockdown of atp6v1h was performed in mouse chondrocytes (ATDC5) and osteoblasts (MC3T3). Knockdown was confrmed by qPCR, showing reduced *Atp6v1h* expression as normalized with *Gapdh* (A and D). *Mmp9* (B and E) and *Mmp13* (C and F) in chondrocytes and osteoblasts are slightly reduced after *Atp6V1h* knockdown, suggesting that *Atp6v1h* may not have a profound impact on osteoblasts or chondrocytes.
(TIF)

S9 Fig Analysis of osteoclast maturation by TRAP. Differentiation of Raw264.7 (pre-osteoclast) cell was induced by RANKL (100ng/ml). Raw264.7 was transfected by siRNA anti-ATP6V1H 2 times, and osteoclast cells were identified by TRAP staining (red color). (A) The ATP6V1H siRNA groups have significantly more mature osteoclasts (red staining of large multi-nuclei cells) than the scrambled controls. Included here are sets of five randomly selected fields of images of ATP6V1H siRNA groups and scrambled controls. (B) The ratio of TRAP positive cells and total cells is significantly higher in ATP6V1H siRNA group. Specific data is listed in S2 Table.
(TIF)

S10 Fig Analysis of osteoclast by TRAP in adult zebrafish. Adult zebrafish (10 month old) of +/- for *atp6v1h* (line *atp6v1h*^*la439*^) and wild type sibling were fixed, sectioned and stained by TRAP. *Atp6v1h*^*la439+/-*^ fish appear to have more TRAP staining compared to their sibling (n = 3, two images are shown here for wild type and +/- mutant). Scale bar: 50 um.
(TIF)

S1 Table Table lists the primer sequences used in qPCR analysis and generation of probes for *in situ* hybridization. (DOCX)

S2 Table Cell counts of TRAP-negative and positive Raw cells induced by RANKL. (DOCX)

S1 Methods This section describes the methods used to generate supplementary figures in the study. (DOCX)
