# Correction: ATP6V1H Deficiency Impairs Bone Development through Activation of MMP9 and MMP13

**DOI:** 10.1371/journal.pgen.1006624

**Published:** 2017-02-27

**Authors:** Yihan Zhang, Haigen Huang, Gexin Zhao, Tadafumi Yokoyama, Hugo Vega, Yan Huang, Raman Sood, Kevin Bishop, Valerie Maduro, John Accardi, Camilo Toro, Cornelius F. Boerkoel, Karen Lyons, William A. Gahl, Xiaohong Duan, May Christine V. Malicdan, Shuo Lin

The Funding section is incorrect. The correct funding information is as follows: This work was supported in part by the Intramural Research Program of the National Human Genome Research Institute and the National Natural Science Foundation of China (81470728, XD), Chinese Scholarship Council (YZ) and Science and Technology Program of Shenzhen (JCYJ20151030170755264). The funders had no role in study design, data collection and analysis, decision to publish, or preparation of the manuscript.

In the Author Contributions section, Xiaohong Duan (XD) should be listed under Funding Acquisition after WAG.
