# Supplementary figures and images for: ATP6V1H Deficiency Impairs Bone Development through Activation of MMP9 and MMP13
Source: PLoS Genet. 2017 Feb 3;13(2):e1006481. doi: 10.1371/journal.pgen.1006481 (PMC5291374; doi:10.1371/journal.pgen.1006481)

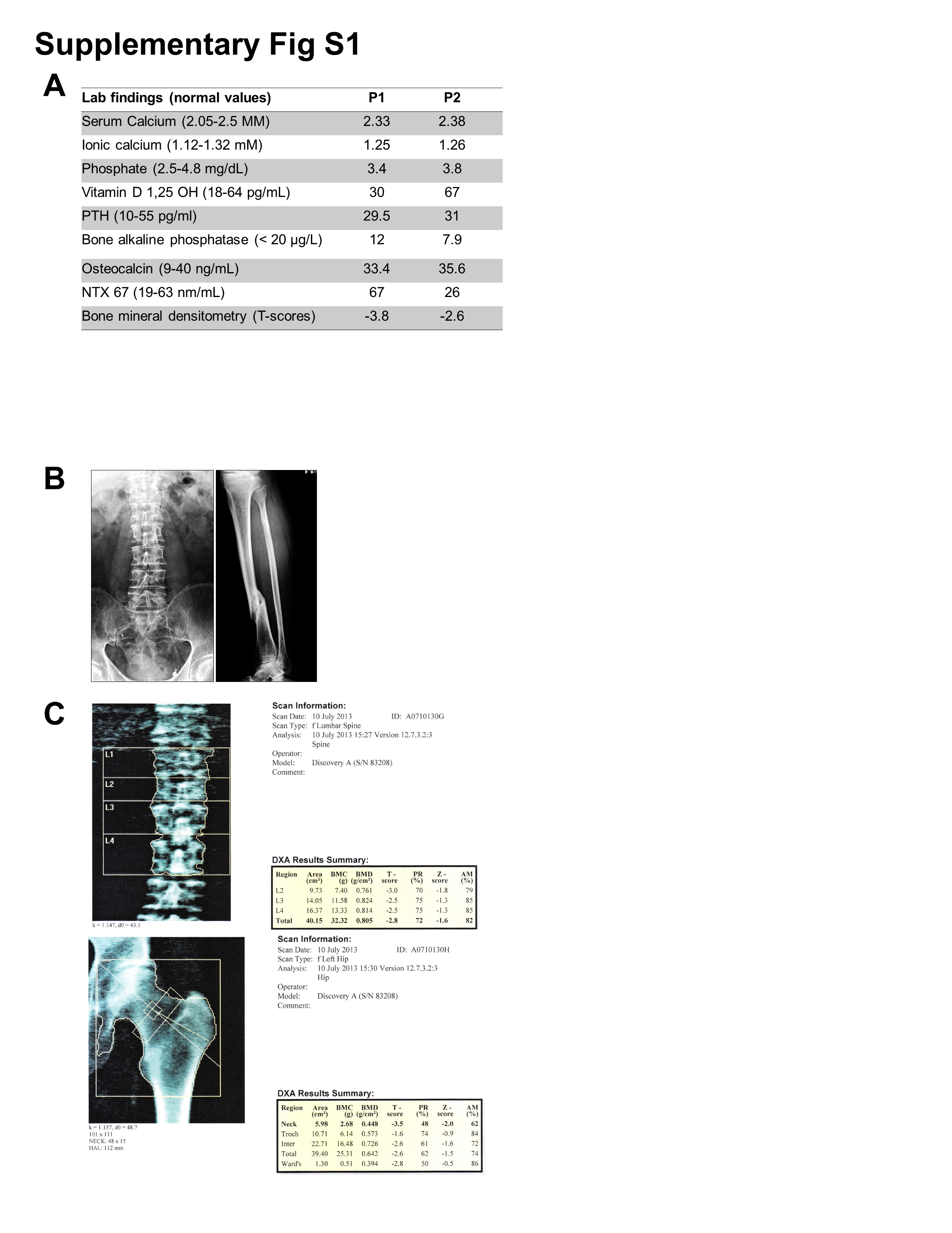

Supplement: S1 Fig — ATable shows the levels of biochemistry findings from Patient 1 (P1) and Patient 2 (P2), and Bone mineral densitometry scores.BRadiographic pictures from I.2 (father of P1) showing scoliosis and fractured fibula.CDual-energy x-ray absorptiometry (DXA) results of I.2. (TIF) [file pgen.1006481.s001.tif]

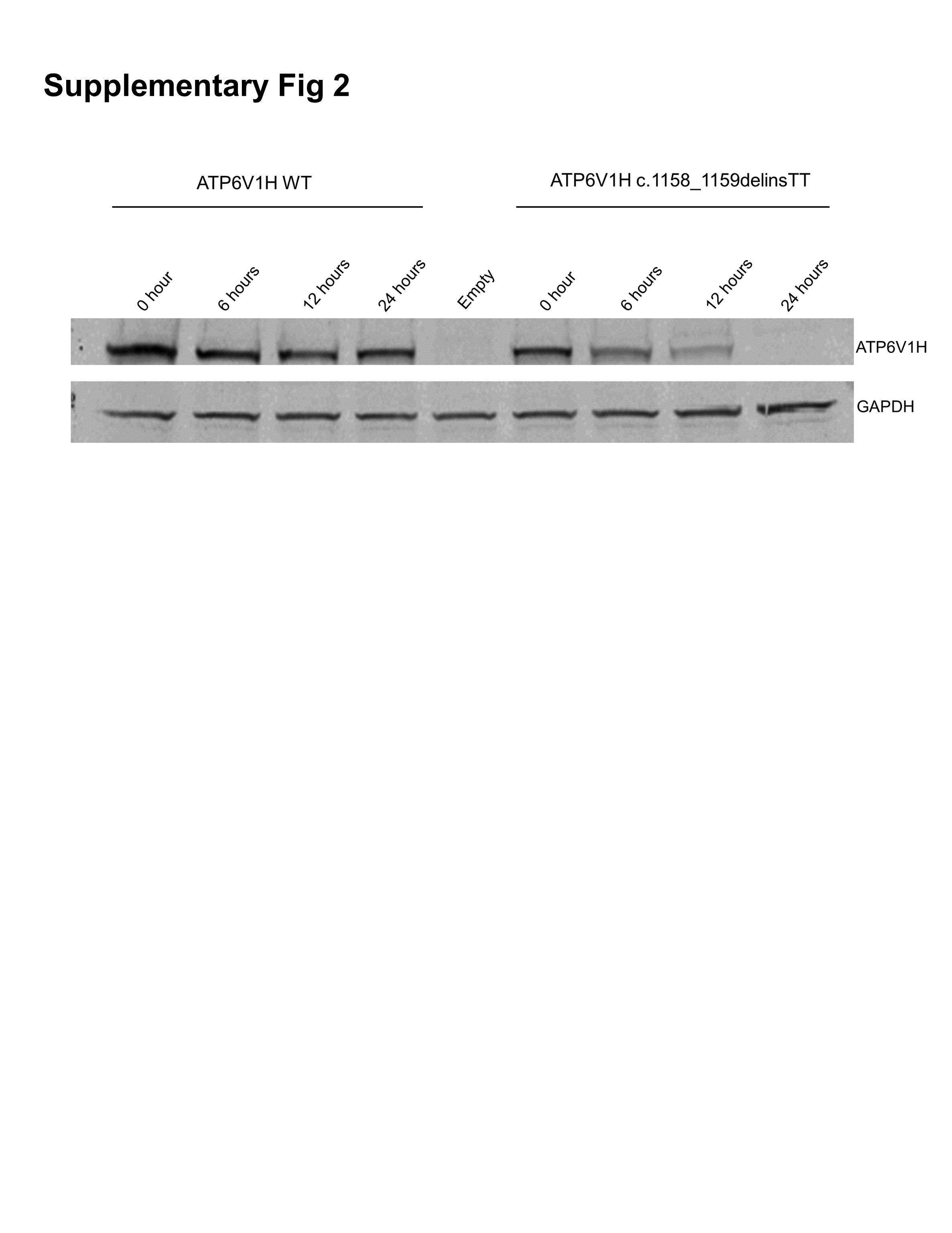

Supplement: S2 Fig — Cell lysates form HEK cells transfected with full length ATP6V1H wild-type or mutant (c.1158_1159delinsTT) cDNA and treated with cycloheximide were subjected to western blot. Figure shows ATP6V1H bands at 0 hour (baseline), 6 hours, 12 hours, and 24 hours. Protein loading is normalized with GAPDH. Experiments were done in three replicates. (TIF) [file pgen.1006481.s002.tif]

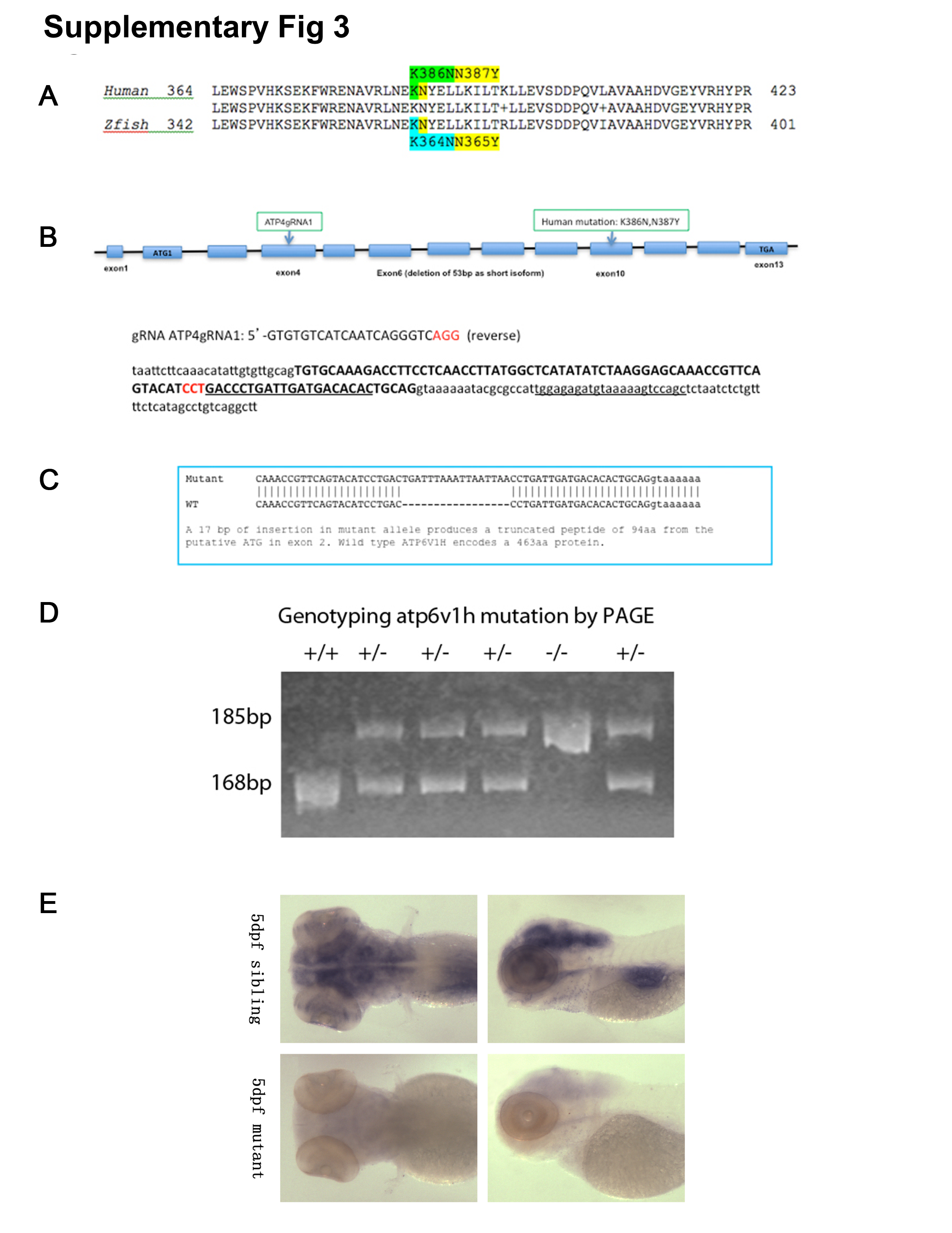

Supplement: S3 Fig — Protein alignment of ATP6V1H in human (NP_057025.2) and zebrafish (NP_775377.1) shows high homology (~85%). More importantly, the region where the mutation is located is highly conserved (A). Using CRISPR/Cas9, guide RNA (gRNA) targeting exon 4 of zebrafish atp6v1h was co-injected with Cas9 mRNA into zebrafish embryos and detected by T7 endonuclease digestion. Sequence of gRNA and target is shown (B). Several founders with indels were screened for germline transmission, and the one with 17bp insertion was used throughout the study; The 17bp insertion is predicted to produce a termination codon 94 amino acids after initiation of start codon (C). Genotyping done by polyacrylamide gel electrophoresis (PAGE) analysis demonstrating a 168 bp band for wild type allele, and 185 bp band for mutant allele; representative results for wild type (+/+), heterozygous (+/-), and homozygous (-/-) embryos are shown (D). Using probes specific for atp6v1h, RNA in situ hybridization analysis of atp6v1h showed expression in the head region in wild type embryos, while the expression is nearly absent in homozygous atp6v1h embryos, suggesting that the 17bp insertion may lead to nonsense-mediated decay (NMD) of mRNA and result in loss-of-function of this gene (E). (TIF) [file pgen.1006481.s003.tif]

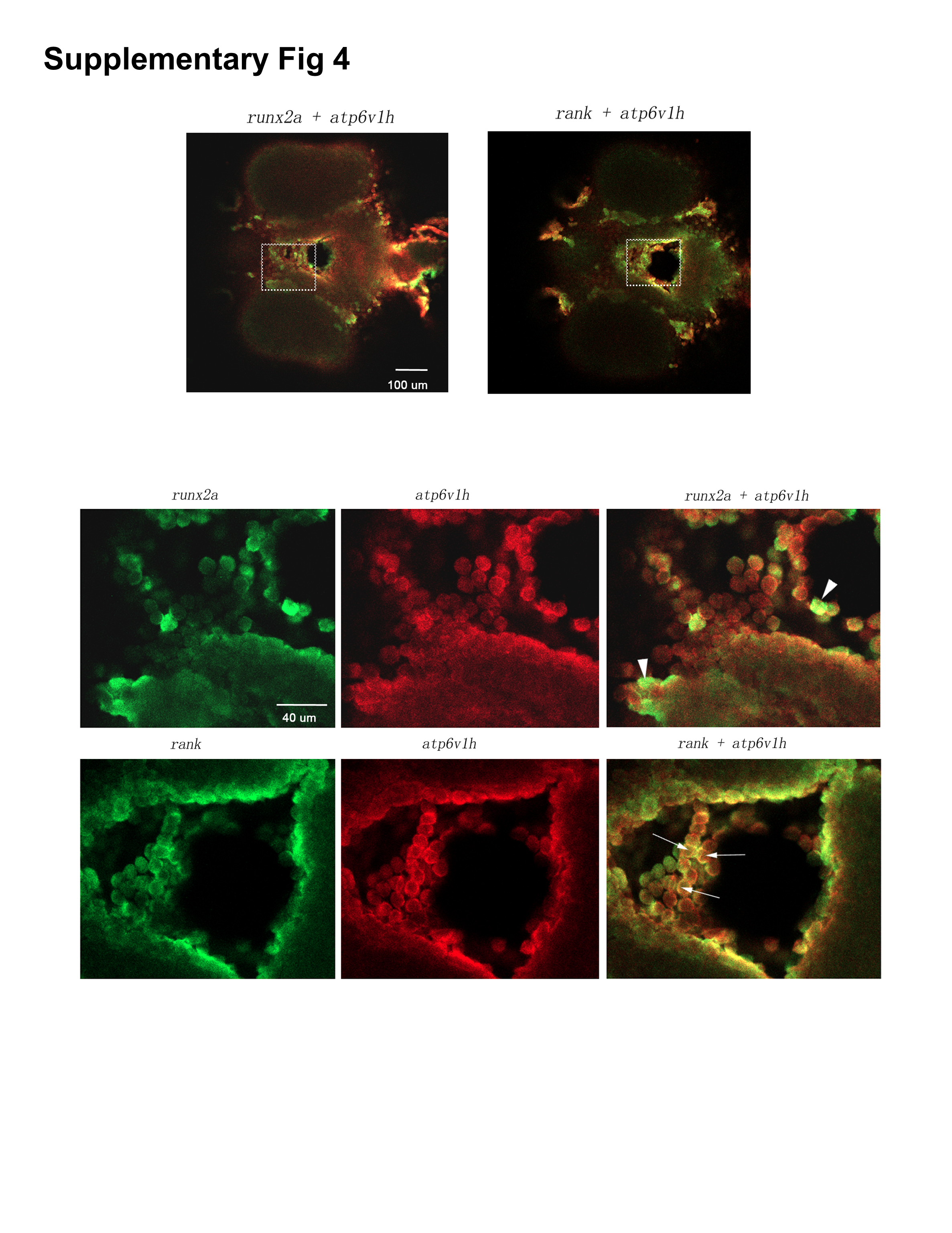

Supplement: S4 Fig — Double fluorescence RNA whole mount in situ hybridization with atp6v1h and rank or runx2a probes were performed on 60 hpf embryos and imaged by confocal microscopy. Upper panels are ventral views of merged lower magnification images of runx2a and atp6v1h or rank and atp6v1h, in which the boxes indicate the regions shown in the lower panels of higher magnification. The lower panels are images of individual colors (green fluorescence for probes of runx2a or rank, red fluorescence for atp6v1h) and merged color (runx2a + atp6v1h, rank+ atp6v1h). Arrowheads point to cells that are mainly positive for runx2a and arrows point to cells that appear positive both for rank and atp6v1h (co-expression: yellow fluorescence). (TIF) [file pgen.1006481.s004.tif]

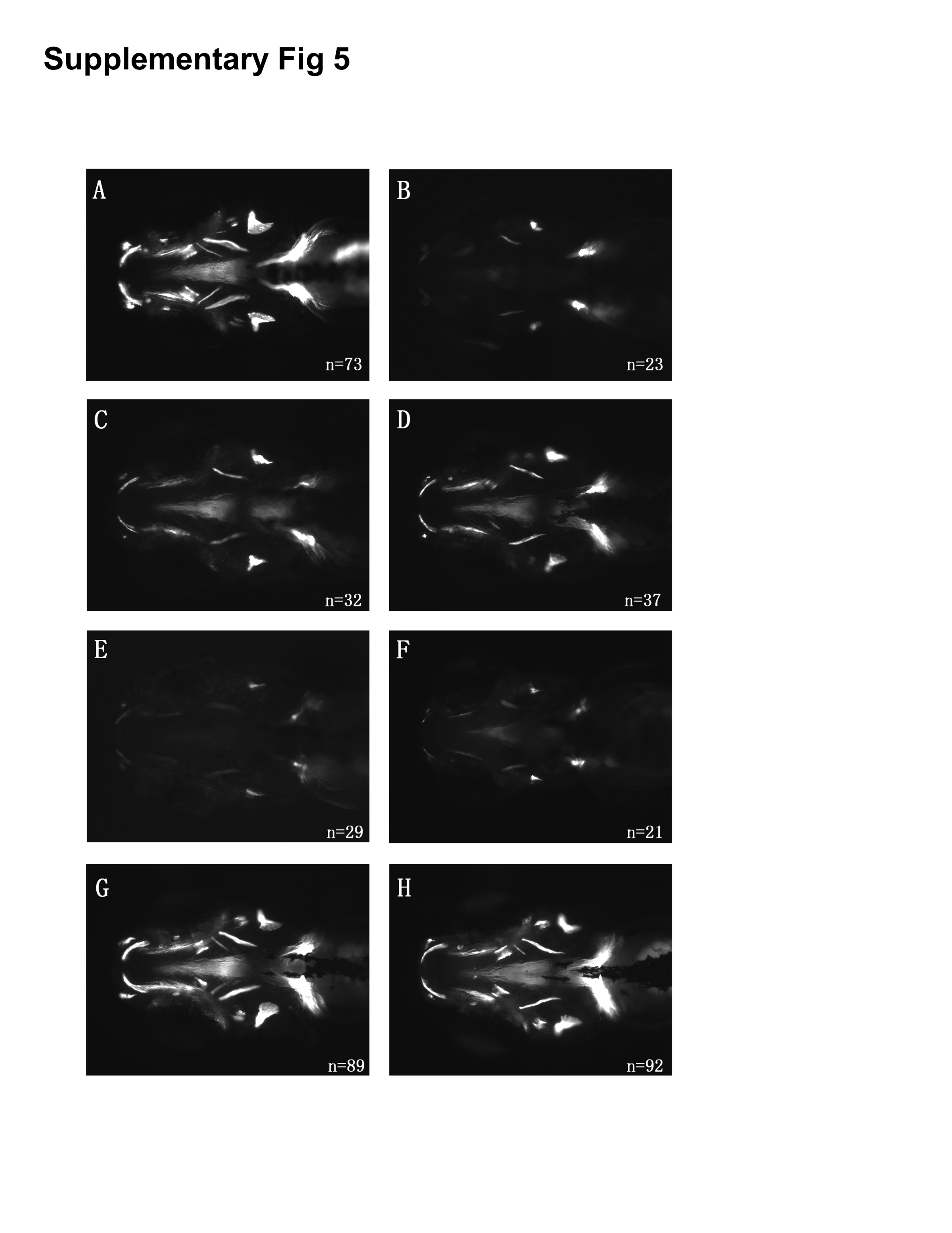

Supplement: S5 Fig — Bone staining of uninjected wild type and atp6v1h -/- embryos; loss of bone mineralization is seen in atp6v1h mutant embryos (A and B). atp6v1h mutant embryos at single-cell stage injected with wild type mRNA (C, 200 pg; D, 300 pg) were stained at 5dpf; representative figures demonstrate the rescue of bone phenotype, seen as increase in bone mineralization. atp6v1h mutant embryos injected with mutant mRNA containing the same K386N and N387Y mutations found in humans (E, 200 pg; F, 300 pg) at single-cell stage and stained at 5 dpf; representative figures show the absence of bone staining, indicating that the mutations create a non-functional gene. Wild type and mutant mRNA were injected into wild type embryos at single-cell stage and stained at 5 dpf (G and H); representative figures show no alteration in morphology and bone staining, suggesting the absence of gain-of-function resulting from overexpression of either wild type or mutant mRNA. (TIF) [file pgen.1006481.s005.tif]

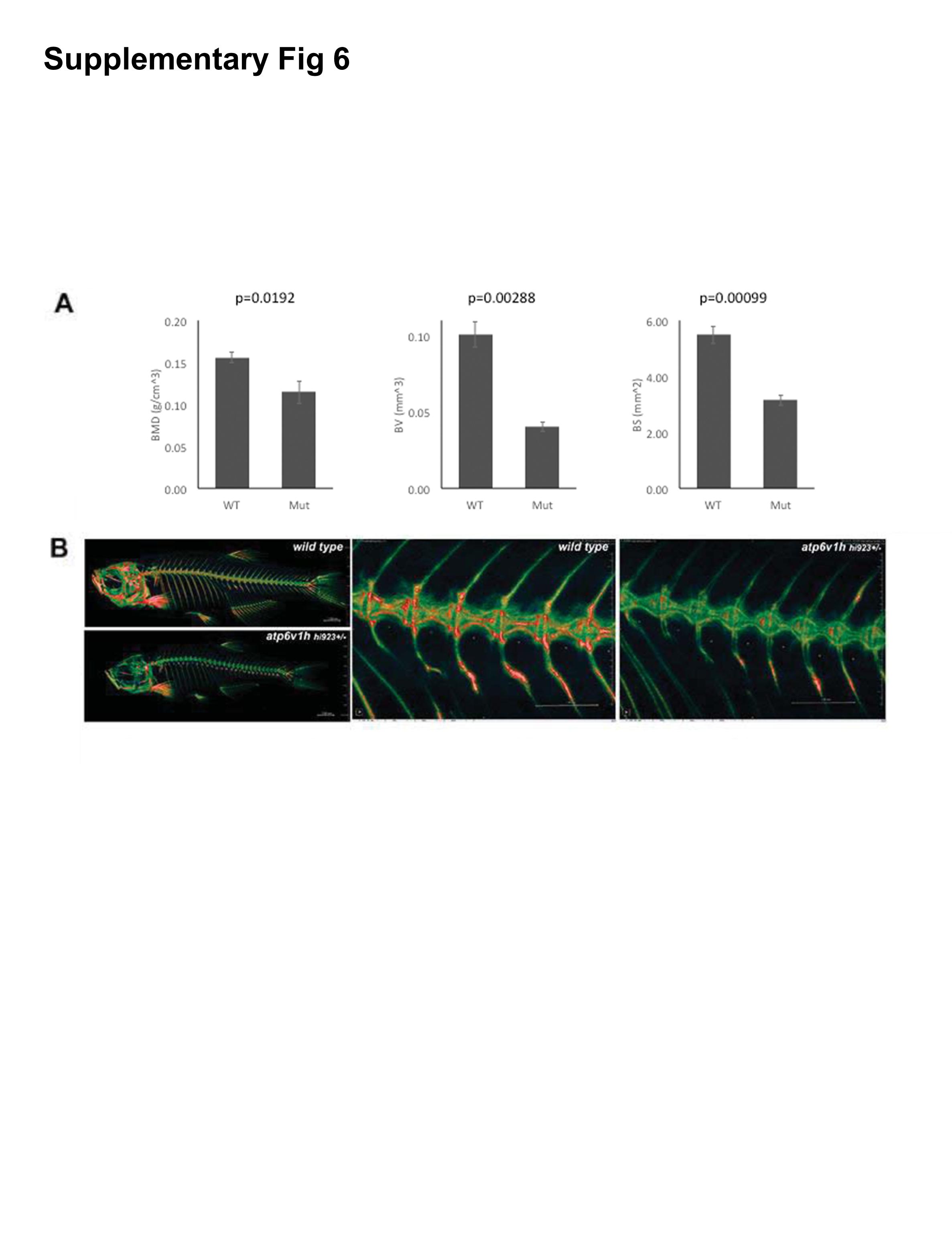

Supplement: S6 Fig — A. Micro-CT data of the first to fifth caudal vertebrae. Wild type sibling +/+. Heterozygous +/-. BMD, BV and BS designate bone mineral density, bone volume and bone surface (n = 3). B. Micro-CT images of bone in 8-month-old male adult wild type zebrafish and heterozygous allele of retrovirus insertion mutant, atp6v1hhi923+/- (n = 5 each). (TIF) [file pgen.1006481.s006.tif]

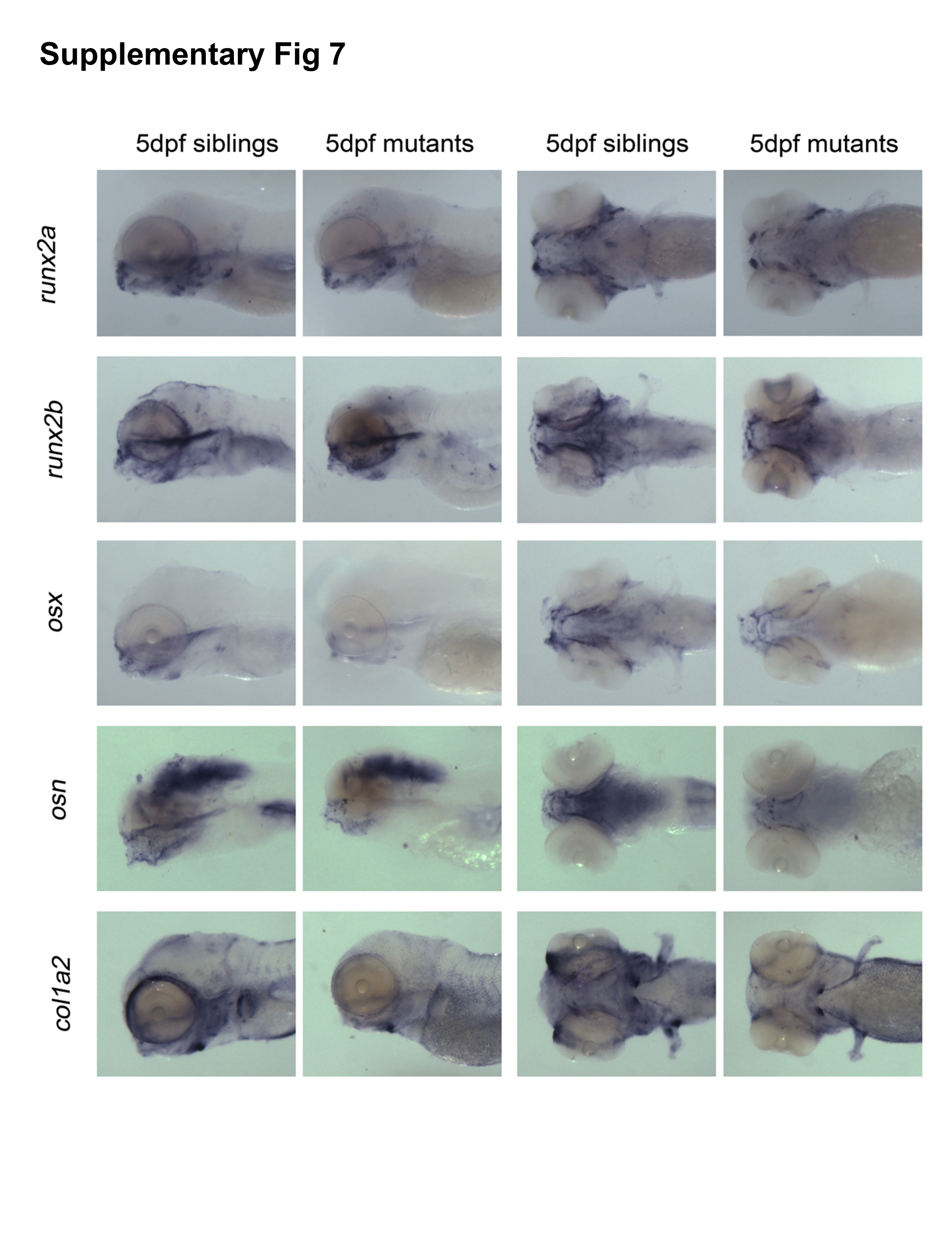

Supplement: S7 Fig — In situ hybridization markers of osteoblast development (runx2a, runx2b, osx, osn, col1a2) show slight reduction between 5dpf siblings (wild type) or mutant (-/-). (TIF) [file pgen.1006481.s007.tif]

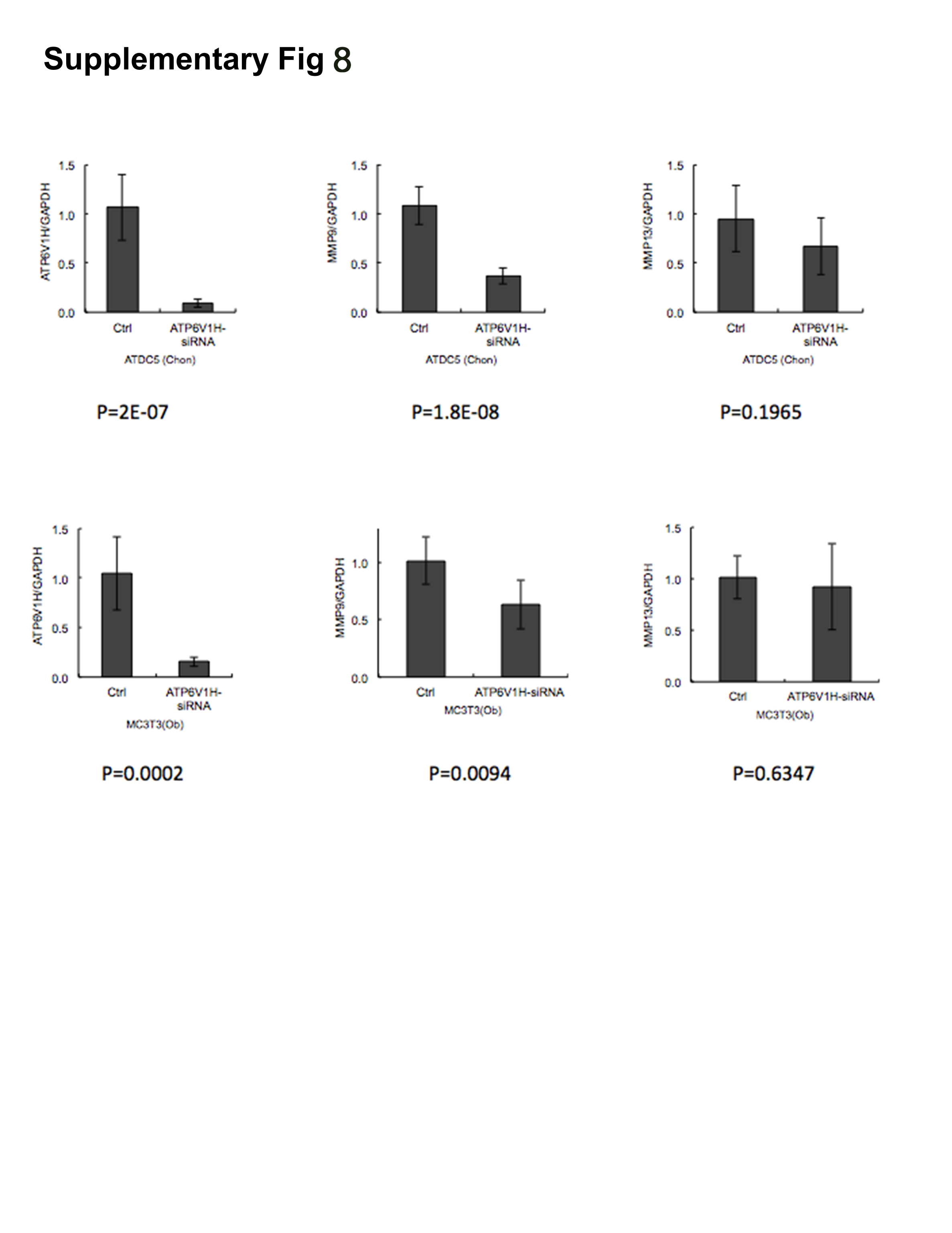

Supplement: S8 Fig — siRNA knockdown of atp6v1h was performed in mouse chondrocytes (ATDC5) and osteoblasts (MC3T3). Knockdown was confrmed by qPCR, showing reduced Atp6v1h expression as normalized with Gapdh (A and D). Mmp9 (B and E) and Mmp13 (C and F) in chondrocytes and osteoblasts are slightly reduced after Atp6V1h knockdown, suggesting that Atp6v1h may not have a profound impact on osteoblasts or chondrocytes. (TIF) [file pgen.1006481.s008.tif]

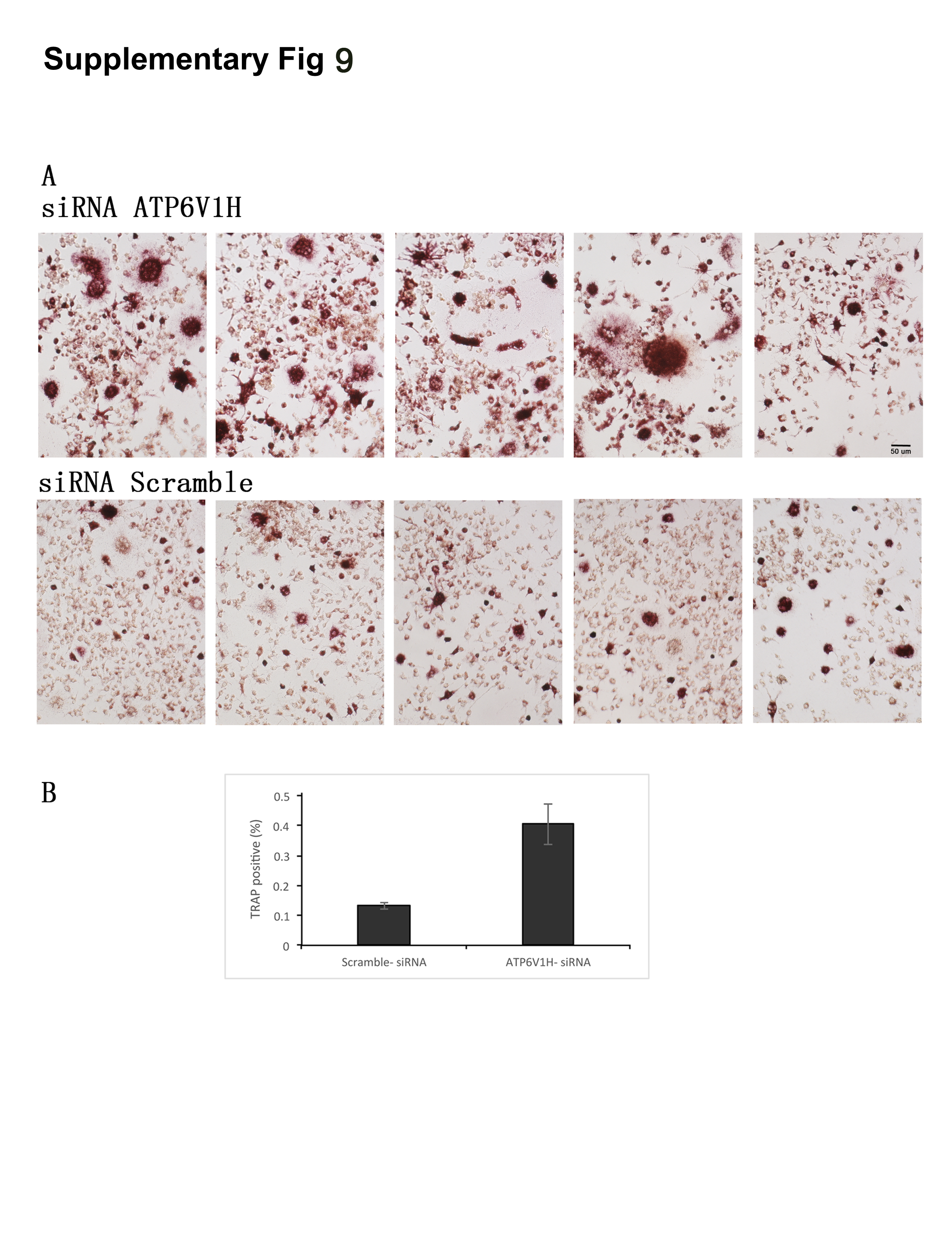

Supplement: S9 Fig — Differentiation of Raw264.7 (pre-osteoclast) cell was induced by RANKL (100ng/ml). Raw264.7 was transfected by siRNA anti-ATP6V1H 2 times, and osteoclast cells were identified by TRAP staining (red color). (A) The ATP6V1H siRNA groups have significantly more mature osteoclasts (red staining of large multi-nuclei cells) than the scrambled controls. Included here are sets of five randomly selected fields of images of ATP6V1H siRNA groups and scrambled controls. (B) The ratio of TRAP positive cells and total cells is significantly higher in ATP6V1H siRNA group. Specific data is listed in S2 Table. (TIF) [file pgen.1006481.s009.tif]

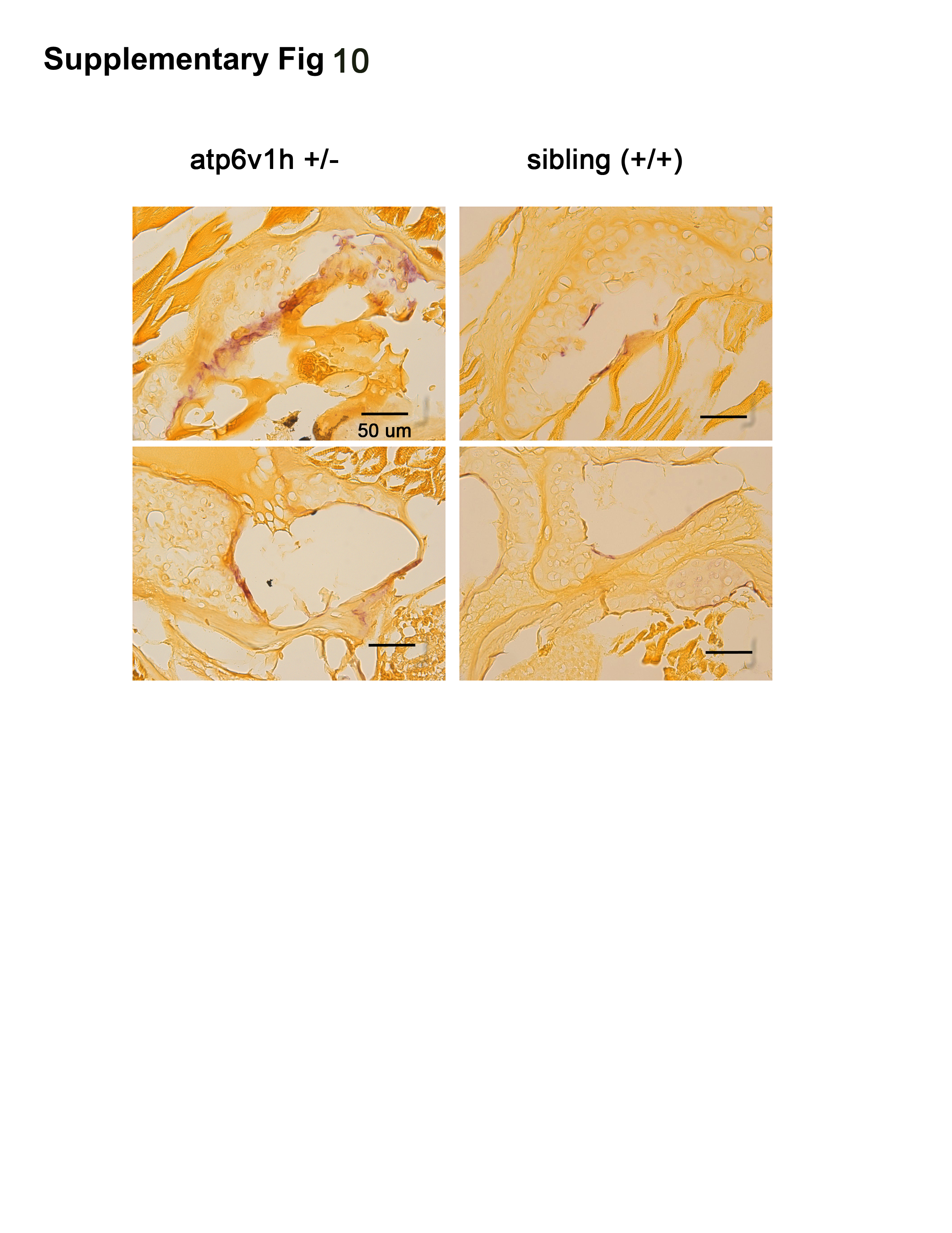

Supplement: S10 Fig — Adult zebrafish (10 month old) of +/- for atp6v1h (line atp6v1hla439) and wild type sibling were fixed, sectioned and stained by TRAP. Atp6v1hla439+/- fish appear to have more TRAP staining compared to their sibling (n = 3, two images are shown here for wild type and +/- mutant). Scale bar: 50 um. (TIF) [file pgen.1006481.s010.tif]
