# Supplementary material for: ATP6V1H Deficiency Impairs Bone Development through Activation of MMP9 and MMP13
Source: PLoS Genet. 2017 Feb 3;13(2):e1006481. doi: 10.1371/journal.pgen.1006481 (PMC5291374; doi:10.1371/journal.pgen.1006481)
Supplement: S1 Table — (DOCX) [file pgen.1006481.s011.docx]

**Supplemental Information**

**S1_Table. PCR Primers Used for Analyzing Osteoblast/Osteoclast Gene Markers and ATP6V1H Mutation.**

|  | | **Forward Primers** | **Reverse Primers** |
| --- | --- | --- | --- |
| **qPCR** | ***runx2a*** | **5'-GACCATGGTGGAGATCATAGC** | **5'-GGGTTCGTGAATACTGTGATTG** |
|  | ***runx2b*** | **5'-AGAGCTTCACCCTGACGATTAC** | **5'-AGGTACGATGGGTATGTCTGGT** |
|  | ***osx*** | **5'-CGCTCAATCCTCAAATGCCG** | **5'-TTTACCGTACACCTTCCCGC** |
|  | ***osn*** | **5'-GTGGACGTTAAGAGCGGGAA** | **5'-GAGGACGACACTGGACAAGG** |
|  | ***osc*** | **5'-ATCAGCTGACACAGAAGCGA** | **5'-GGCGGTGATGATTCCAGACG** |
|  | ***col1a2*** | **5'-TGCAGTGACTTCGTACCTAGC** | **5'-CACCAATAGCTCCAGCCTGT** |
|  | ***alp*** | **5'-ACGGAGAGGAAACACAACTG** | **5'-TTCATTGGGCATGTCTGCAT** |
|  | ***rank*** | **5'-GCCTGACTACATGACCATCA** | **5'-GACATTCGTCCTGACTGCTG** |
|  | ***ctsk*** | **5'-TGATGACAGAGTGGGAAAGTTG** | **5'-TCCTGAAGTGTTGTAGGCACAC** |
|  | ***mmp9*** | **5'-CATCCGCAACTACAAGACATTC** | **5'-GGTCCAGTATTCATCGTCATCA** |
|  | ***mmp13a*** | **5'-GTGATGAAAAAGCCCCGCTG** | **5'-CATCGTCGAAATGAGCGTCG** |
|  | ***mmp13b*** | **5'-AACCACAAACGTGACCTTCA** | **5'-CGCAACCAGGAACAGATTGA** |
|  | ***mmp2*** | **5'-CAAACTTTTGCTGCGCCCT** | **5'-CAGATCTGGGGTGTGCCCTAA** |
|  | **β-actin** | **5'-ATGCCCCTCGTGCTGTTTTC** | **5'-GCCTCATCTCCCACATAGGA** |
|  | | | |
| ***in situ***  **cDNA probe** | ***runx2a*** | **5'-AAAGAGCTTCACGCTGACAATCAC** | **5'-GAGGCGCCGTAGTAGAGATATG** |
|  | ***runx2b*** | **5'-GAAGAGCTTCACCCTGACGATT** | **5'-AGGAAGCACCGTAATAGAGATA** |
|  | ***osx*** | **5'-TTAGACATGACGCATCCTTACG** | **5'-GGTTAAATCTCCAGCAGTCCAC** |
|  | ***osn*** | **5'-GTGGACGTTAAGAGCGGGAA** | **5'-TTTGCCATCGCGGTAAGAGT** |
|  | ***col1a2*** | **5'-TGCAGTGACTTCGTACCTAGC** | **5'-CCTTCACTCCAACAGGTCCG** |
|  | ***rank*** | **5'-ACTGGGACGTTTTCAGACCG** | **5'-CACTCTCCTGCACCGACTTC** |
|  | ***mmp9*** | **5'-CACAGCTAGCGGATGAGTATCTGAAGC** | **5'-AATGGAAAATGGCATGGCTCTCC** |
|  | | | |
| **genotyping** | ***atp6v1h*** | **5'-GTTGCAGTGTGCAAAGACCT** | **5'-CTGACAGGCTATGAGAAAACAGA** |
