# Supplementary material for: ATP6V1H Deficiency Impairs Bone Development through Activation of MMP9 and MMP13
Source: PLoS Genet. 2017 Feb 3;13(2):e1006481. doi: 10.1371/journal.pgen.1006481 (PMC5291374; doi:10.1371/journal.pgen.1006481)
Supplement: S2 Table — (DOCX) [file pgen.1006481.s012.docx]

**S2 Table. Cell counts of TRAP-negative and positive Raw cells induced by RANKL.**

|  | Scramble-siRNA-1 | Scramble-siRNA-2 | Scramble-siRNA-3 | Scramble-siRNA-4 | Scramble-siRNA-5 | **Average** | ATP-siRNA-1 | ATP-siRNA-2 | ATP-siRNA-3 | ATP-siRNA-4 | ATP-siRNA-5 | **Average** |
| --- | --- | --- | --- | --- | --- | --- | --- | --- | --- | --- | --- | --- |
| TRAP-negative | 200 | 279 | 248 | 248 | 327 | **260.4** | 117 | 128 | 145 | 166 | 199 | **151** |
| TRAP-positive | 30 | 46 | 43 | 36 | 44 | **39.8** | 83 | 114 | 115 | 114 | 82 | **101.6** |
| TOTAL | 230 | 325 | 291 | 284 | 371 | **300.2** | 200 | 242 | 260 | 280 | 281 | **252.6** |
| Ratio | 0.13 | 0.14 | 0.15 | 0.13 | 0.12 | **0.13** | 0.42 | 0.47 | 0.44 | 0.41 | 0.29 | **0.41** |
