# Supplementary material for: ATP6V1H Deficiency Impairs Bone Development through Activation of MMP9 and MMP13
Source: PLoS Genet. 2017 Feb 3;13(2):e1006481. doi: 10.1371/journal.pgen.1006481 (PMC5291374; doi:10.1371/journal.pgen.1006481)
Supplement: S1 Methods — (DOCX) [file pgen.1006481.s013.docx]

**Supplemental Information**

**S1_Methods**

***Generation and Genotyping of ATP6V1H Mutation in Zebrafish***

The zebrafish ATP6V1H protein shares over 85% amino acid identity compared to the human ATP6V1H (Fig S2). To target the ATP6V1H gene in zebrafish using CRISPR/Cas9, we designed gRNA of 5’-GTGTGTCATCAATCAGGGTCAGG targeting the fourth exon of *atp6v1h*. Injection of the synthesized gRNA along with Cas9 mRNA into the zebrafish embryos successfully targeted the genomic site, as detected by T7 endonuclease 1 digestion. F1 embryos from injected founders were screened for germline transmission by the same method and several lines carrying indels were identified. We selected one with a 17 bp insertion for studies in this paper. This insertion would produce a truncated peptide of 94aa, whereas the wild type ATP6V1H encodes a 463aa protein. This mutation in individual embryos can be genotyped using the two primers outside the insertion listed in Table S1 by polyacrylamide gel electrophoresis (PAGE) analysis. We confirmed that embryos (N>100) with the bone phenotype were genotypically -/- mutant and embryos (N>100) with no phenotype were +/- or +/+ using this method. More importantly, we were able to rescue the phenotype by injection of wild type mRNA of *atp6v1h*. These analyses confirmed that the bone defect is caused by a genetic mutation in *atp6v1h*.

***Analysis of protein stability***

HEK293 cells (ATCC®) were grown on non-coated cell culture dishes and grown in high-glucose (4.5 g/L) DMEM supplemented with 10% FBS and penicillin-streptomycin. At ~70% confluence, cells were transfected with a mammalian expression vector (pDEST31) containing the full length *ATP6V1H* wild-type or mutant (c.1158_1159delinsTT) cDNA using Lipofectamine 2000 (Invitrogen). Twenty four hours after transfection, cells were treated with 50 µM cycloheximide (Sigma-Aldrich Corp.), and harvested at different time points (baseline or 0 hour, 6 hours, 12 hours, 24 hours), were lyzed with RIPA lysis buffer (50mM Tris, pH 7; 150 mM NaCl; 0.1% SDS; 0.5% sodium deoxycholate; 1% Triton x-100; 1mM EDTA) supplemented with protease inhibitors (complete, Mini, EDTA-free, Roche), and were subjected to immunoblotting. Samples were electrophoresed on a 3-8% Tris-Glycine gel and blotted onto a 0.2 µm nitrocellulose membrane (Invitrogen). Membranes were blocked with PBS-T, supplemented with 5% non-fat milk, washed and incubated with primary rabbit anti-human ATP6V1H (Abcam, ab67125) and GAPDH (Santa Cruz, sc-32233) antibodies, followed by appropriate IRDye 680RD or IRDye 800CW-conjugated secondary antibodies (Li-Cor Biosciences). Proteins were detected using the Li-Cor imaging system (Li-Cor Biosciences). The molecular weight ladder used was the Precision Plus Protein™ Kaleidoscope™ Standards (Bio-Rad) or the HiMark™ Pre-stained Protein Standard (Life Technologies)

***siRNA knockdown experiments in mouse cells***

Dulbecco’s modified eagle medium (DMEM) and fetal bovine serum (FBS) were provided by GIBCO (Grand Island, NY, USA). Recombinant murine sRANKL was purchased from Shenandoah Biotechnology, Inc. (Warwick, PA, USA) . Leukocyte Acid Phosphatase 5, tartrate resistant (TRAP) kit (#387A-KT) was obtained from Sigma-Aldrich Inc. (St. Louis, MO, USA). siRNA against ATP6V1H (Cat#M-063917-01) was purchased from DharmaconTM (Lafayette, CO, USA).

RAW264.7 murine monocyte/macrophage cells (#TIB-71) were obtained from the American Type Culture Collection (ATCC, Rockville, MD, USA). Cells were maintained in DMEM supplemented with 10% heat-inactivated FBS and antibiotics, streptomycin (100 μg·mL−1), and penicillin (100 µg·mL−1). Cells were incubated at 37 °C in a humidified atmosphere with 95% air and 5% CO2.

RAW264.7 cells were seeded into a 24-well plate at 2 × 10^4^ cells/well in DMEM containing 10% heat-inactivated FBS in the presence of 100 ng·mL−1 sRANKL for 6 days to induce osteoclastic differentiation. Medium and all factors were replaced on day 3. At the end of culture, cells were fixed with 3.7 % formaldehyde in PBS and stained using a TRAP staining kit according to the manufacturer's instructions. Large, TRAP-positive cells containing multinuclear were mature osteoclasts. Photomicrographs were taken with a Nikon Ti-DH Microscope.

At the end of culture, total RNA was extracted and reverse transcribed to cDNA using Power SYBR® Green Cells-to-Ct™ Kit (Ambion, Foster City, CA, USA). Thermo Scientific Maxima SYBR Green/ROX qPCR Master Mix(2X) was used for the quantitative real-time PCR assay. Analysis of relative gene expression was performed using the 2−ΔΔCT method and results were normalized to the housekeeping gene (GAPDH). All the primers used in the study were synthesized by Valuegene Inc. (San Diego, CA, USA) and are listed in Table 1.

Statistical analyses were performed on datasets from three independent experiments, and are represented as the mean ± standard deviation (SD). Statistical significance between experimental and control groups was directly compared by a Student t test to establish significance at the p < 0.05 level.
